# On the Usage of Linear Regression Models to Reconstruct Limb Kinematics from Low Frequency EEG Signals

**DOI:** 10.1371/journal.pone.0061976

**Published:** 2013-04-17

**Authors:** Javier M. Antelis, Luis Montesano, Ander Ramos-Murguialday, Niels Birbaumer, Javier Minguez

**Affiliations:** 1 Aragon Institute of Engineering Research (I3A), University of Zaragoza, Zaragoza, Aragon, Spain; 2 Institute of Medical Psychology and Behavioral Neurobiology, Eberhard-Karls-University, Tubingen, Germany; 3 TECNALIA, Health Technologies, San Sebastian, Basque Country, Spain; 4 Ospedale San Camilo-Istituto di Ricovero e Cura a Carattere Scientifico, Venezia, Lido, Italy; 5 BitBrain Technologies, Zaragoza, Spain; Florey Institute of Neuroscience & Mental Health, Australia

## Abstract

Several works have reported on the reconstruction of 2D/3D limb kinematics from low-frequency EEG signals using linear regression models based on positive correlation values between the recorded and the reconstructed trajectories. This paper describes the mathematical properties of the linear model and the correlation evaluation metric that may lead to a misinterpretation of the results of this type of decoders. Firstly, the use of a linear regression model to adjust the two temporal signals (EEG and velocity profiles) implies that the relevant component of the signal used for decoding (EEG) has to be in the same frequency range as the signal to be decoded (velocity profiles). Secondly, the use of a correlation to evaluate the fitting of two trajectories could lead to overly-optimistic results as this metric is invariant to scale. Also, the correlation has a non-linear nature that leads to higher values for sinus/cosinus-like signals at low frequencies. Analysis of these properties on the reconstruction results was carried out through an experiment performed in line with previous studies, where healthy participants executed predefined reaching movements of the hand in 3D space. While the correlations of limb velocity profiles reconstructed from low-frequency EEG were comparable to studies in this domain, a systematic statistical analysis revealed that these results were not above the chance level. The empirical chance level was estimated using random assignments of recorded velocity profiles and EEG signals, as well as combinations of randomly generated synthetic EEG with recorded velocity profiles and recorded EEG with randomly generated synthetic velocity profiles. The analysis shows that the positive correlation results in this experiment cannot be used as an indicator of successful trajectory reconstruction based on a neural correlate. Several directions are herein discussed to address the misinterpretation of results as well as the implications on previous invasive and non-invasive works.

## Introduction

Brain-Machine Interfaces (BMI) have emerged as a new alternative to recover functionality in impaired limbs, where the neural signals related to movement are mapped onto the multidimensional control of a physical effector. Hitherto, 2-D movement control achieved with EEG in humans is very similar to that achieved with cortical neurons [1], [2], while 3-D movement control has been achieved with EEG in humans [3], [4], and with cortical neurons in monkeys [5], [8]. Most recent development in humans includes a subject with tetraplegia [9]. Previous studies of movement control, whether using spikes or EEG, involve task-specific adaptations of the brain to evoke changes in the brain oscillations used in the BCI decoding process and from which the user receives feedback. Thus, these results do not necessarily indicate whether the signals recorded during imagined or normal muscle-based control contain information about the limb kinematics. People may learn to use EEG features to control multi-dimensional movements even though normal EEG does not contain detailed limb kinematic information (i.e. full reconstruction of 2D or 3D trajectories). Therefore, it is still not clear whether this type of information is present in the EEG. Indeed, EEG signals were believed to lack sufficient signal-to-noise ratio and bandwidth to encode detailed movement kinematics [10]. This assumption has been challenged in recent years generating a vivid discussion in the field [11], [12]. Using low frequency EEG, reconstruction of hand movement profiles have been reported (e.g., position and velocity profiles in 2D [13], [14] and 3D work-spaces [15]–[19]). These results indicate that detailed limb kinematic information could be present in the low frequency components of EEG, and could be decoded using linear regression models. However, there is dubiety regarding the effectiveness and performance of the applied methods [20], [21].

This paper analyzes the mathematical implications of the use of linear regression methods in the reconstruction of limb trajectories using neural temporal signals as well as of the use of the correlation as the main metric to evaluate the decoding. The two key mathematical results are related to: 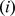 the use of a linear regression model to adjust two temporal signals (neural signal and limb kinematics) imposes that both signals must span the same frequency range, independent of the nature and information content of the signals; and 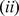 the use of correlation to evaluate the fitting of two trajectories could lead to overly-optimistic results, as this metric is invariant to scale and has a non-linear nature that leads to higher values for sinus/cosinus-like signals at low frequencies. These two properties may result in an misinterpretation of the results of the reconstruction, likely to be present when the signal to be predicted only contains low frequencies. This is the case in the reconstruction of limb kinematics, as the typical experimental settings result in velocity profiles similar to low frequency sinusoidal signals 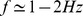. Indeed, the first mathematical result justifies why the only frequency range of the temporal signal (e.g. EEG) suitable for the reconstruction is low frequency. The second property states that a given positive correlation value is not an absolute indicator of reconstruction accuracy. Thus, the crucial question is whether there is a neural correlate in this low frequency EEG or the reconstruction results are due to a misinterpretation of the analysis.

To address this issue, seven subjects participated in an experiment, performing self-selected and self-initiated 3D hand reaching movements towards predefined targets, while EEG and hand position (and velocity by numerical integration) were simultaneously recorded. The chance level of the reconstruction was empirically obtained by shuffling the recorded data (i.e., randomly assigning recorded EEG signals to velocity profiles) and by using randomly generated synthetic data. Although the accuracy of the reconstruction results were in line with studies that reported the multidimensional limb kinematics reconstruction from low frequency EEG, a systematic analysis revealed that the reconstruction results were at the chance level. The present study suggests caution when selecting linear models and corresponding evaluation metrics to address the reconstruction of limb trajectories from low frequency temporal neural signals. Results could be due to an inherent misinterpretation of the results of the analysis and not due to a unique and significant relationship between limb velocity profiles and low frequency EEG activity. For future EEG decodings, the authors recognize the need to use a validation procedure similar to the methodology proposed in this paper to prove the real effectiveness and congruency between EEG activity and limb trajectory reconstruction.

## Mathematical Analysis of the Methods

### Linear regression models for time series

Let 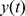 be the variable of interest at time 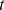 and let 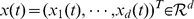 be a vector of potential predictor variables at time 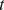. A linear regression models the response 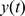 as a linear function of 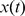:
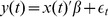
(1)where 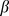 are the linear parameters and 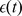 is the error term. If needed, a bias term can be incorporated into the predictor 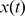. This general model varies depending on the input variables 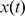. The simplest model involves only data at time 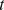, denoted a static regression. In the context of neural signals, it is common to include lagged variables for the predictor variables, i.e., 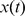 contains information from previous points in time, which is denoted as auto-regressive models (AR). Despite the fact that AR models present dependencies among variables, ordinary linear regression models still provide a reasonable solution under the assumptions of stationarity and weak dependence. The use of a linear regression model with temporal signals presents some properties in terms of the frequencies of the input and output variables that affect the decodings:

#### Property 1

Let SC be the spectral content of a temporal signal, then:
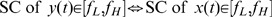
(2)

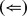
 If the predictor variables 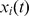 present the spectral content (SC) within a frequency range 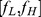, then all the spectral content of the predicted signal 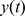 is within this frequency range 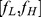. This can be easily shown by the Fourier transforms 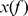 and 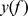 of the time series defined by 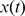 and 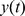. Using the linear properties of the Fourier transform [22], the model can be expressed in the frequency domain as 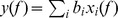 where 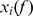 is the Fourier transform of the predictor variable 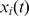. Let 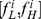 be the frequency band for 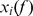 and let 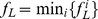 and 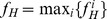 be the minimum and maximum frequencies among all predictors. Then, any linear combination of the signals 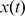 will result in a 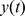 signal confined to the 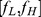 frequency band (as the model coefficients 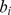 influence the amplitude and phase of the predicted 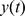 but do not affect the oscillation frequency). 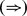 When using a linear regression model for the estimation of a time series 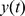 with the spectral content confined to a band 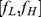, 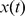 must have spectral content in 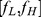 (in fact, this content is the only one useful for the adjustment). This is straightforward from the previous property. Note that if 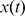 does not have spectral content in 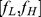, then it is not possible to estimate 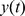 with this model.

The consequence of this property in the decoding of limb kinematics (denoted by 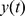) from neural temporal signals such as the EEG (denoted by 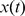) follows:

#### Consequence 1

On one hand 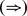, given a limb velocity profile 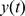 with frequencies confined to a band 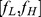, only neural signals 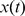 with spectral content in 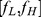 will contribute to predict 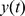. Temporal signals with frequency content out of this band may have an adverse effect on the fitting as they are noise for the regression process. In addition, a change in the frequency range of the limb velocity 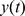 to 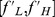 will also change the useful frequency range of neural signal 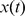 for the fitting to that band, regardless of whether there is a neural correlate within this band. On the other hand 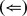, filtering the neural signal 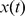 at 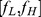 implies that only velocity profiles 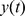 with spectral content at 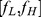 can be reconstructed.

In practical terms, this consequence implies that, if both signals are related by a linear regression, there will not be a predetermined frequency range in the neural signals 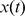 to decode the limb kinematics 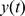 (i.e., if the neural signal spectra used in the reconstruction are confined to a specific band, only velocity profiles with frequencies within this band could be reconstructed, and if the limb frequency of motion changes then the neural signal spectra used in the reconstruction must also change).

### Evaluation metrics

The metric that captures the similarity between two signals in decoding studies is the linear correlation 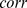 between the measured signal 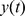 and the corresponding predicted one 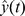. The 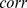 metric has the following two properties:

#### Property 2

The 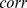 of two signals 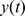 and 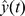 is invariant to the scale of the data:
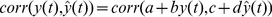
(3)with 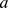, 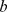, 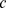, and 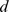 constants and 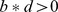. For two signals 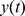 and 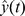, the computation of their correlation is not affected by transforming 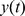 to 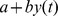 and/or 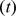 to 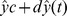.

#### Property 3

Let be 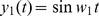 and 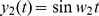 two sinusoid signals with 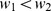, then:
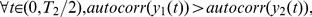
(4)where 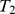 is the period of 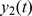. The autocorrelation function 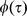 of a sinusoid with unitary amplitude and frequency 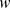 is 
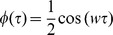
. Then, as 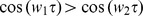 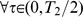 it follows that 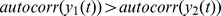. The same result is valid for cosines signals.

The consequences of both properties in the evaluation of the decoding are:

#### Consequence 2

As the correlation is invariant to scale, a high correlation between the velocity measurement 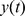 and the velocity estimation 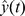 does not necessarily indicate an accurate estimation of the limb trajectory (i.e., low position error) as the position is the integral of the velocity in time.

#### Consequence 3

The correlation of sinusoid/cosinus signals with equal amplitudes and small time-shifts is higher at low frequencies. As the time frequency profile of limb velocity in a center-out task is similar to the shape of a sinusoid (start with zero velocity, a progressive acceleration and finally deceleration until zero at the end point), then the correlation between the real 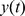 and the estimated velocity profiles 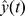 will be less sensitive to temporal shifts at lower frequencies.

In practical terms, both consequences imply that positive values of correlation between real 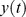 and estimated velocity profiles 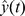 do not necessarily imply a correct trajectory reconstruction. This effect is more likely to occur when the signals under evaluation are sinusoid-like at low frequencies, which is the case of natural limb motion in center-out tasks.

### Example of the model properties

**Figure 1:**
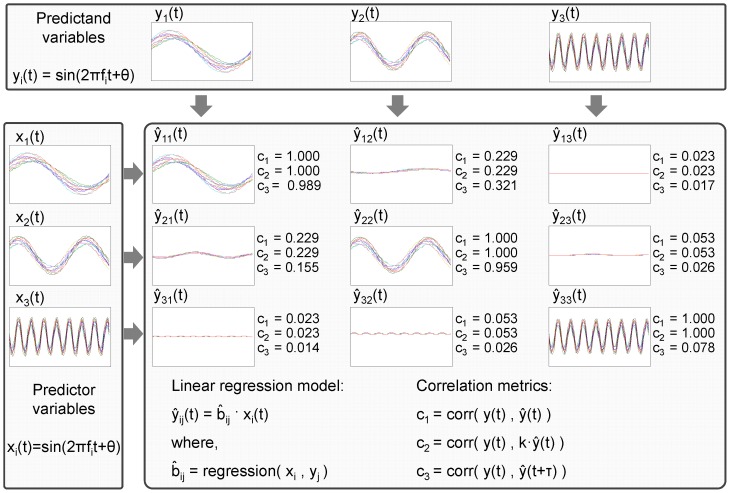
Illustration of the model and metric properties. The left panel shows three datasets of temporal signals 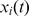 representing predictor variables at 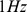, 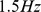 and 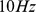. The upper panel shows the predictands variables 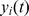, which are identical to the predictors (i.e. they correspond to a linear model 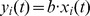 with 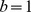). Each dataset contains 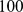 signals and 90% of them was used to train a linear regression model while the remaining 10% was used to evaluate performance. The linear regression model was used predict each dataset from itself and the others. For each case, the reconstructed signals and correlation results are shown in the middle panel. The effect of the artifact is revealed in the usage of the regression model and correlation to validate datasets 1 and 2, where the correlation values are approximately 0.23, despite having different frequencies.

This subsection describes, through an example, how these properties may lead to a misinterpretation of the decoding results. The example estimates three datasets of predictand sinusoids at different frequencies, where the first two datasets present similar frequencies while the third one is at a frequency one order of magnitude higher (1, 1.5 and 10 Hz respectively). For the sake of simplicity, each predictand was estimated from the three datasets, that is, from itself and from the other two predictors which are at different frequencies (see Fig. 1 for further details). For each combination of predictor and predictand, the example provides the correlation between the original predictand and the linear regression reconstruction (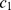 in Figure 1), and between a scaled (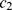) and a temporally shifted (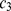) version of the reconstructed signal.

The results are displayed in Figure 1. The reconstructed signals are equal to the real signals when both datasets are the same, while they differ in magnitude and frequency when the datasets are not equal (i.e. when they do not agree in frequency, as explained by property 1). It is also relevant to mention that the frequency of reconstructed datasets is equal to the predictor dataset irrespective of the frequency of the predicted dataset, i.e., only frequencies that agree with the predictors can be obtained with a linear model. Since the example is noise free, the reconstruction for the correct frequency is perfect and 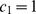. As the frequency between predictor and predictand increases, the correlation decreases (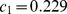 between the first and second datasets with a frequency difference of 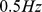, and 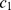 drops to zero between the third and the first two datasets when frequency differs in one order of magnitude). In all cases the results for 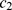 are the same than for 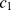. The same correlation results are obtained irrespectively of the scale of the reconstructed variables, explained by property 2. When the datasets agree, 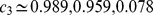 for the first, second and third dataset, respectively. Time-shifts in the reconstruction reduce the correlation with a larger effect on the dataset with higher frequencies, explained by property 3.

Note that the effect of the combination between the properties of the linear regression and the correlation metric may lead to a misinterpretation of the reconstruction: for the first and second datasets, the fact that they are sinusoids-like signals with low and similar frequencies leads to a correlation of 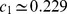 (which could be interpreted as a positive decoding result). However, the reconstruction is poor and far from the original signal (see 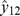 and 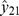). At low frequencies, the correlation values can even increase when shifting the signals, as shown by the value of 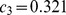 for 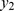 and 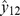.

## Methods

The experimental design follows the experiment and analysis described in [15], [17], [19] and is extended to understand whether there is a neural correlate behind the decoding of the limb velocity profile using EEG signals and a linear regression model.

### Data recording and reaching apparatus

#### EEG system

EEG activity was recorded by a gTec system (2 synchronized gUSBamp amplifiers), with 28 electrodes according to the 10/10 international system, with the ground on FPz and reference placed on the left earlobe. Vertical and horizontal EOG were also recorded. EEG and EOG signals were acquired with a sampling frequency of 256Hz, power-line notch-filtered and lowpass-filtered at 60 Hz.

#### 3D Motion capture system

The 3D limb position was recorded by a video-based VICON motion capture system, which recorded 3D positions of 22 visual reflective markers attached to the body (head, torso, shoulder, arms, wrists, hands, and fingertips). The sampling frequency of the device was 100 Hz.

#### Reaching apparatus

**Figure 2:**
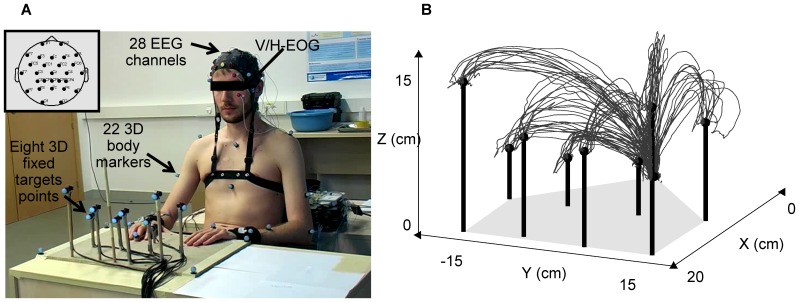
Experimental design. (A) Snapshot of the experimental setup showing a participant with the EEG electrodes (electrode locations are shown in the upper left of the picture), the visual reflective markers attached to the body, and the reaching apparatus. The participant has given written informed consent to publication of their photograph. (B) Examples of recorded trajectories for the hand of one subject during the reaching operations towards the target locations.

The apparatus presented 9 positions in a 3D workspace (size 20–30–15 cm), with one position used as homing location for the finger and the others as locations to reach in the workspace (minimum and maximum distances from the homing location to any location were 10 and 30 cm, respectively). These locations were equipped with reflective markers for the establishment of the 3D location by the VICON, and with electric switches for the synchronization between the onset and the termination of the movement by the EEG and VICON simultaneously, by means of a common electric signal (Figure 2A).

### Experimental design

Seven right-handed male healthy volunteers participated in the experiments (age range: 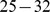 years) after the protocol was approved by the Institutional Review Board of the University of Zaragoza. All participants were asked to read and sign an informed consent form to participate in the study. The participants were seated in a comfortable chair in front of the reaching apparatus (Figure 2A). Participants were instructed to move the right arm-hand-finger from the homing location to a self-chosen location (center-out paradigm) and then return to the homing position. This process was self-paced, lasting on average 7.5 s (minimum 2.8 s and maximum 9.7 s). During the reaching task, subjects were asked to maintain a natural and constant posture and to minimize blinking while maintaining gaze fixed at a reference point in the center of the apparatus. For the remaining time they were allowed to blink and rest. During the experiment the participants performed reaching operations towards fixed target locations on the apparatus. The experiment was executed in five time blocks of 5 minutes each, where the subjects executed in mean 200 reaching operations towards eight locations (minimum 128 and maximum 262). Figure 2B illustrates the trajectories recorded for subject 1. All trials were epoched from 500 ms prior to the movement onset until the end of the reaching movement.

### EEG and Movement Data Pre-Processing

EEG data were re-sampled to 100 Hz and re-referenced by a CAR montage. The two most frontal sensors were excluded from the analysis to mitigate the influence of any ocular artifact. A total of 26 sensors were then used. All EEG traces were visually inspected and non-satisfactory or noisy trials were discarded. The movement-related power spectra changes at different frequency bands of the artifact-free EEG activity were examined with time-frequency analysis. The EEG was divided into epochs of 500 ms of length and windowed with a Hamming function (frequency resolution of 2 Hz) with successive steps of 25 ms before applying the Fourier transform. The relative power spectra changes between rest and movement were computed as the ratio between the power spectra of a baseline at each frequency bin (mean power spectra in the time interval from −1s to −0.6s to the movement onset) and the spectra of the pre-movement and movement for each trial (time interval from −0.4s to 1s relative to movement onset).

Motion data were visually inspected and non-satisfactory or noisy trials were discarded. The last 100 ms of all trajectories were also eliminated as in some trials participants slightly moved their finger to push the switch, leading to high frequency position artifacts. Data were smoothed using a moving average filter (window size of eight samples) and re-referenced to the homing point. The velocity profiles were then calculated by numerical integration of each limb position profile and standardized by subtracting the mean and dividing by the standard deviation.

### Linear Decoding Model

The linear decoding model computes the relationship between the movement kinematics and the EEG data. EEG data were filtered by a zero-phase shift, sixth-order, low-pass Butterworth filter with a cutoff frequency of 1Hz, and then the standardized temporal difference was computed for each electrode, following [15], [17], [19], [23]:
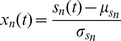
(5)where 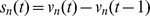 is the difference in time of the EEG sensor 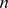, and 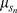 and 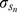 are the mean and standard deviation of the temporal difference. Although many variants of the linear regression model of Equation (1) can be developed, a common approach is to use an autoregressive model to decode each dimension separately [15], [17], [19], [23]–[26]:
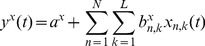
(6)

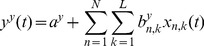
(7)

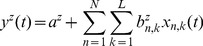
(8)where 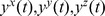 are the hand velocities in the X, Y and Z dimensions, 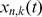 is the standardized difference in time of the EEG at electrode 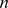 and time lag 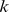 (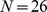 is the number of electrodes and 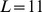 is the number of time lags corresponding to 100ms of EEG activity prior to time 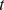). The model parameters 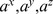 and 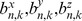 were estimated using Multiple Linear Regression (MLR).

### Metrics and Evaluation Process

The metrics used to assess the goodness of the reconstruction were the Pearson correlation coefficient (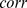) [13], [15], [23], [24] and the normalized root mean square error (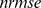) [27]–[29] between the measured and reconstructed velocities, which are in line with previous non-invasive and invasive studies. The distributions of these metrics were characterized by the median and the 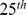 and 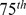 percentiles. The outliers (i.e., values that do not belong to the distribution) were identified as values greater than 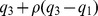 or smaller than 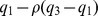, where 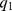 and 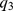 are the 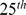 and 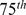 percentiles, respectively. If data are normally distributed, 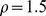 corresponds to approximately 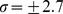 and 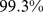 coverage.

The relative contribution of each electrode in each time lag was computed in terms of the magnitude of the regression coefficients 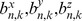:
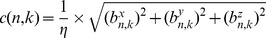
(9)where 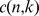 is the relative contribution of electrode 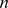 at time lag 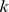, 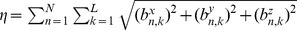 is a normalization factor, 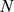 is the number of sensors, and 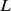 is the number of time lags. In order to examine the contribution of different scalp regions in the decoding model, the relative contribution of the electrodes was computed as:
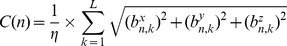
(10)


The contribution of the different time lags in the decoding model was computed as:
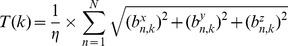
(11)


The performance of the decoding model was evaluated by a 10-fold cross-validation procedure (to avoid over-fitting due to the relatively small number of trials compared to the dimension of the problem). In this procedure, the full set of trials (a trial is the EEG and velocity profile data of a complete reaching movement) were sampled without replacement to create training and test sets of each fold. To maintain independency of the test and training, all the pre-processing steps that involve multiple trials were computed independently for each fold using only the corresponding training examples. The values of the metrics were computed for each test trial for each fold, and the validation procedure was considered complete when all the ten combinations of training and test data were exhausted.

### Source Localization and Source Contribution

The standardized low-resolution brain electromagnetic tomography source localization method (sLORETA) [30] was employed to localize the brain regions that generate the EEG signals. The contribution of the neural sources in encoding hand kinematics was computed by the correlation values (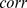) between the time series of the squared activity for each EEG sensor with the time series of the estimated neural sources [15]. The set of 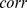 values of each source was multiplied by the regression coefficients of their associated sensors (at the time lag with the highest percentage of contribution, see Equation 9) and the maximum was selected as the contribution of each source in the decoding model. These values were projected onto axial MRI slices of the brain to obtain the brain structure and Brodmann area associated with the sources with the maximum contribution.

### Analysis of Low Frequency EEG Decoding

This section analyzes the computation of the decoding model when low EEG frequencies were used.

#### Evaluation of whether the decoding model performance is above chance level

Five different combinations of EEG measurements and velocity profiles where evaluated to study the performance of the linear decoding model and to test whether the performance is above chance level.

The first combination is the one of interest (Recorded data) since it uses the low frequency recorded EEG and the recorded velocity profiles. The remaining four combinations were used to test the chance level, and there is no association between the EEG and the velocity profiles. In the second combination, the same linear model was applied to a shuffled version of the EEG data (Shuffled data), i.e. velocity profiles were assigned randomly to the EEG of other trials. The shuffling process and computation of the decoding was repeated 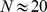 times (per participant) to avoid chance effects due to the stochastic nature of the process (with N  =  10% of the number of training trials). The rationale is to disassociate velocity and EEG information when building the decoding model. Since there may still be some information despite the random association of velocity profiles and EEG measurements, the third combination uses artificially generated EEG measurements and artificially generated velocity profiles (Random EEG&VEL), and the fourth and fifth combinations use recorded (random) EEG measurements with artificial (recorded) velocity profiles (Random EEG and Random VEL). The rationale in these combinations is to evaluate the decoding by using either random EEG and/or random velocity data with no association information between them. The randomly generated EEG measurements and velocity profiles were created using the first- and second-order statistical properties of the original low frequency EEG and velocity profiles. EEG voltage for sensor 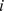 and trial 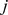 was computed by 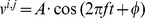, where 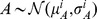, 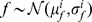 and 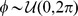. The mean and the standard deviation of the frequency (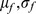) and amplitude (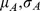) were computed across all trials for each electrode. The velocity profile of trial 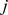 for each coordinate was computed by 
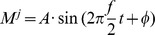
, where 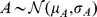, 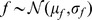 and 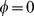. The mean and standard deviation of the frequency (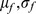) and amplitude (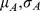) were computed across all trials for each coordinate, and the phase was zero as at 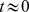 all recorded velocity profiles start to increase from a zero velocity. In this case, there is no information relating the kinematics to the EEG.

#### Progressive Elimination of the Number of Electrodes

Seven decoding models were built in an iterative way, eliminating the electrodes with the greatest contribution to the linear regression model (Equation 10). The number of sensors used in each model was 26, 25, 23, 21, 17, 14 and 11, respectively. The rationale is to eliminate the most prominent neural activity of the decoding before building the next model.

## Experimental Results

### Decoding of kinematics from EEG data

#### Power spectra of the EEG and source localization

**Figure 3:**
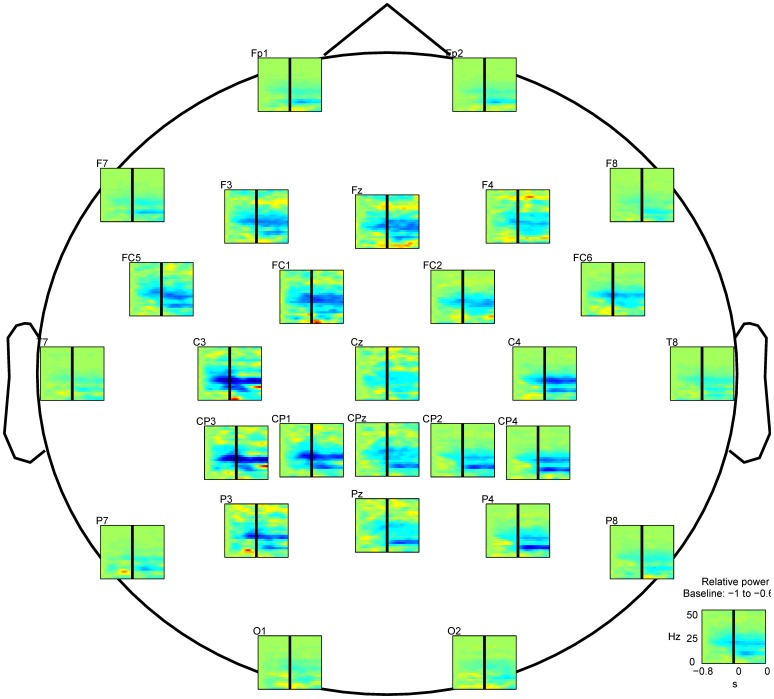
Scalp topography of power spectra changes of the EEG (relative to baseline from −1 to −0.6 s) averaged across all trials and participants. Time in abscissa from −0.8 s to 0.8 s. Frequency in ordinate from 0 to 50 Hz at a resolution of 2 Hz. Movement onset occurs at 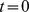 s (solid black line in all graphs). Sensors above the contralateral and ipsilateral motor areas revealed a power increase in the slow wave range (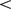
4)Hz and a de-synchronization in the 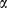 (8-12)Hz and 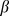 (14-30)Hz frequency bands. Graph at the right lower corner represents the average across-sensors relative power spectra changes of the recorded EEG.

**Figure 4:**
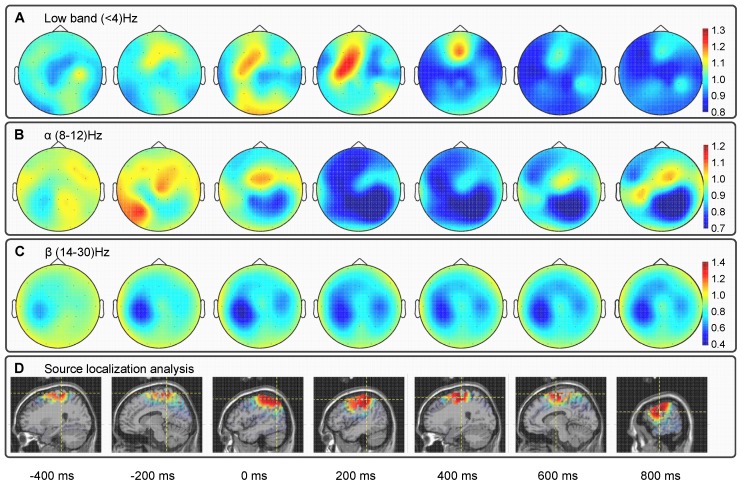
Topographies of changes in the power spectra averaged for all trials and participants (relative to baseline from −1 to −0.6 s with respect to the movement onset) in the (A) 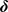, (B) 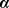 and (C) 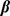 frequency bands. Power increase in the slow wave range started at 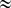
300 ms prior to the movement onset and remained until 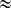
200ms relative to the movement onset. The de-synchronization in the 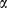 and 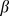 bands started 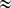
400 ms prior to the movement onset and remained until the end of the movement. (d) The source localization of the underlying EEG activity averaged for all trials and participants revealed a network of activation in the motor-related and neighboring areas prior to the movement onset, and the activation of the contralateral motor cortex during the execution of the movement.

The relative power spectra changes between rest and movement, averaged across all participants, revealed power increase in the slow wave range (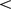
4)Hz and de-synchronization in the 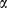 (8-12)Hz and 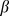 (14-30)Hz frequency bands (Figure 3). Firstly, the power increase is more prominent at sensors located on the contralateral motor and pre-motor scalp and parietal areas (C3, CP3, P3). This power increase started at 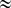
300ms prior to the movement onset in the contralateral parietal areas and then switched to the contralateral motor areas (Figure 4A) at 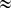
0ms. Secondly, de-synchronization is more prominent in the 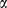 and 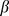 bands of sensors placed on the contralateral (C3, CP3, CP1 and P3) and on the ipsilateral (C4, CP4 and P4) motor and parietal areas. This de-synchronization started 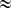
400ms prior to the movement onset and remained until the end of the movement, being less prominent during the pre-movement than during the movement execution (Figures 4B and C). These results are consistent with those of [31] and [32]. The source localization analysis (Figure 4B) revealed the activation of the motor-related (precentral and postcentral gyrus in Brodmann areas 6 and 4) and neighboring brain regions (primary somatosensory cortex in Brodmann areas 1 and 2). Prior to the movement onset (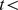
0ms) the cortical activity is distributed in the motor cortex and parietal areas of both hemispheres. During the movement execution (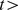
0ms) the cortical activity is estimated with more prominence in the motor areas of the left hemisphere (contralateral to the moved arm).

These results show that different frequency bands are modulated by the motor task performed by the participants, which suggests that evaluation of the decoding model be carried out with EEG activity filtered in those bands.

#### Decoding performance using EEG from different frequency bands

**Figure 5:**
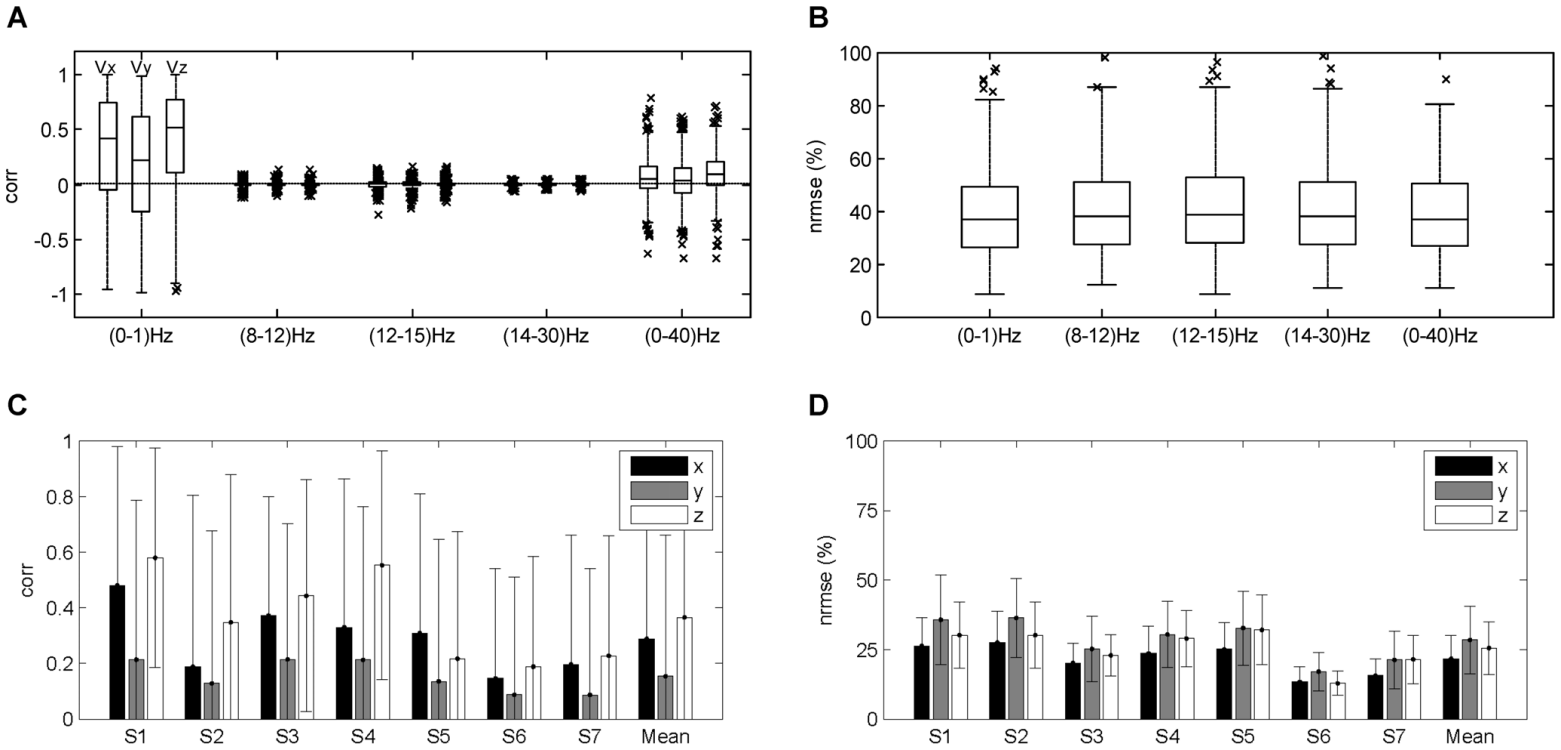
(A,B) Distributions of 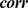 and 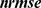 for all participants for decoding models evaluated with EEG in the very low 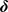 (0–1) Hz, 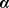 (8–12) Hz, 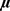 (12,15) Hz, 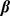 (14–30) Hz and (0–40) Hz frequency bands. In the 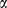, 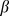 and 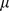 frequency bands, the distributions of 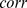 have a significant zero-median distribution in X-, Y- and Z-dimension of the velocity. For the very low 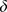 and the (0–40) Hz frequency bands the distributions of 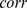 were positive and significantly different from zero, although the medians of the distributions obtained in the very low 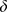 are notably higher than for the (0–40) Hz band. These results support the selection of the very low 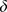 band to further study the decoding of hand velocity. **(C,D) Decoding results using the very low 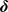 (0–1) Hz frequency band.** mean
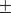
std values of 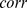 and 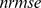 in the decoding of hand velocity profiles using the very low frequency band for all participants plus overall mean.

The decoding model was evaluated with EEG activity filtered in the following frequency bands: very low 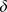 (0-1)Hz, 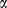 (8–12) Hz, 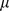 (12–15) Hz, 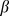 (14–28) Hz and the band (0–40) Hz. Figures 5A and B display the distributions of 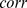 and 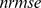 for all trials and participants. With EEG in the 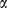, 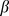 and 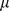 bands, the distributions of 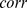 present a zero-median distribution (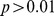, Wilcoxon signed-rank test) in all the dimensions of velocity (X,Y and Z respectively). With EEG in the very low and (0-40)Hz bands, the distributions are positive and significantly different from zero (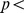
0.01, Wilcoxon signed-rank test). The medians of these distributions obtained in the very low band (0.42, 0.21, 0.52) are one order of magnitude higher than in the (0–40) Hz band (0.05, 0.03, 0.09). The 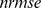 distribution over all participants is not significantly different among bands (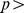
0.05, Kruskal-Wallis test).

Regarding the decoding using the very low frequency band, the averages of the 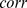 and 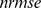 for all participants are displayed in figures 5C and D. The 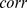 presents positive mean values in all dimensions of the hand velocity, with average 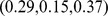. The average of 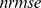 is 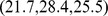, which indicates that the decoding error is on average no greater than 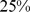 of the trajectory length.

The significant and positive correlation of the very low frequency band, together with the non-significance different from zero and very low correlation in the other bands, are consistent with previous studies [15]–[17]. However, as these results may be misinterpreted (see the mathematical properties described in Section 2), the investigation of whether there is a neural correlate within this band required further analysis.

### Analysis of low frequency decoding results

#### Decoding with shuffled data and/or random EEG and velocity sets

**Figure 6:**
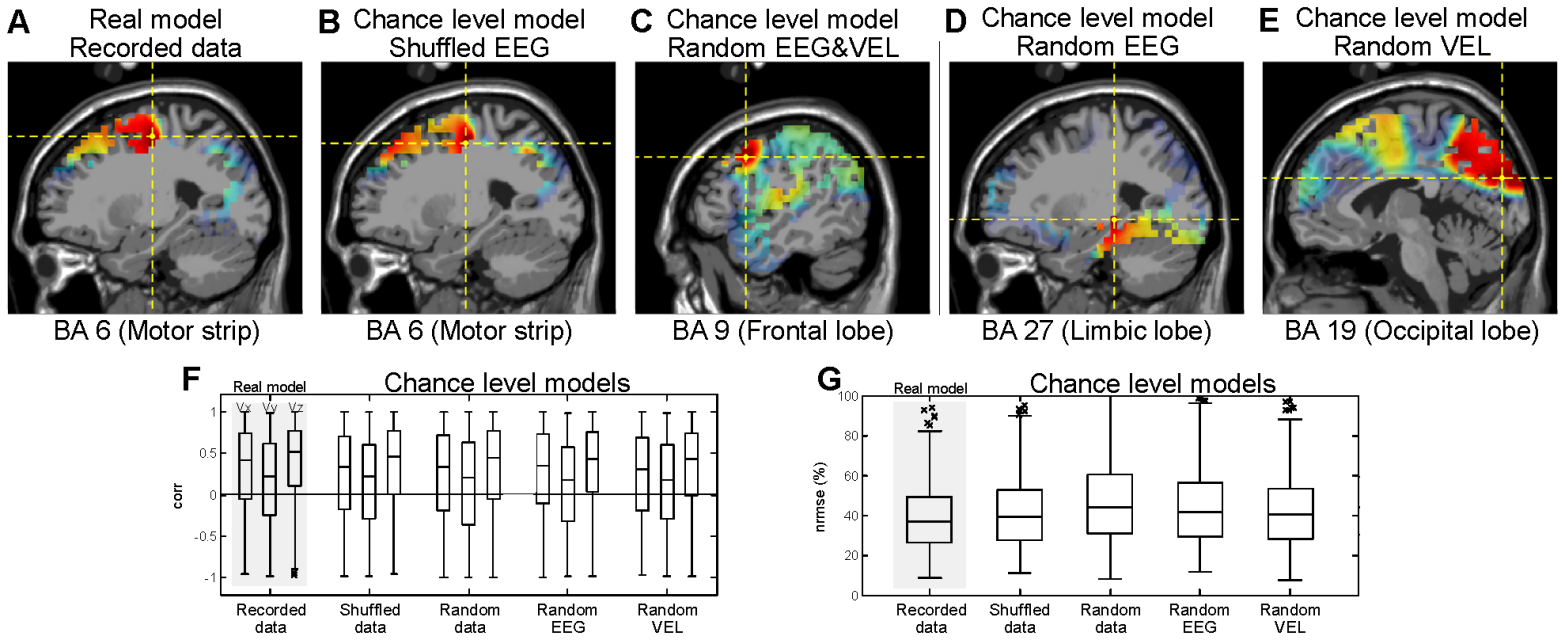
Analysis of low frequency decoding results. (A-E) Neural sources involved in encoding hand kinematic projected onto sagittal MRI slices, with dotted lines indicating the source location with the greatest contribution. Contralateral motor regions of the brain provided the greatest contribution in the decoding models that used (A) recorded and (B) shuffled data. No motor related brain region is involved in the decoding model that used (C) Random EEG&VEL, (D) Random EEG and (E) Random VEL. (F–G) Distributions of (F) 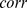 and (G) 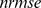 for all participants for the real model (Recorded data) and the chance level models (Shuffled data, Random EEG&VEL, Random EEG and Artificial VEL). These results revealed no significant differences between the real model and the chance level models.

In the decoding models that used recorded and shuffled data, the source contribution analysis showed that the precentral gyrus in the frontal lobe (Brodmann Area 6) of the left hemisphere presented the greatest activation (Figure 6A and B), indicating that the contralateral motor region has the major contribution in limb motion. For the decoding model that used synthetic EEG&VEL, synthetic EEG and synthetic VEL, the medial frontal gyrus in the frontal lobe (Brodmann Area 9), the parahippocampal gyrus in the limbic Lobe (Brodmann Area 27) and the cuneus in the occipital lobe (Brodmann Area 19) presented the greatest activation (Figure 6C, D and E), indicating that the physiologic meaning of these models is not related to the primary motor areas. Note that while the contralateral motor region of the brain was the major contributor in the decoding models built with recorded and shuffled data, this was not the case for the decoding models built with artificial data.

The distributions of 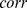 and 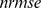 obtained with the real model (Recorded data) and with the chance level models (Shuffled data, Random EEG&VEL, Random EEG and Random VEL) are displayed in Figure 6F and G, for all participants. For each dimension of the velocity profiles, no significant differences were found between the medians of the 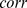 distributions of the real model and the chance level models (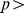
0.01, Wilcoxon rank-sum test). No significant differences were obtained with the distributions of 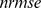 (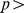
0.01, Wilcoxon rank-sum test) from all the decoding models. These results show that the same performance was achieved regardless of the data used to build the model, that is, the performance of the decoding model is at the chance level.

**Figure 7:**
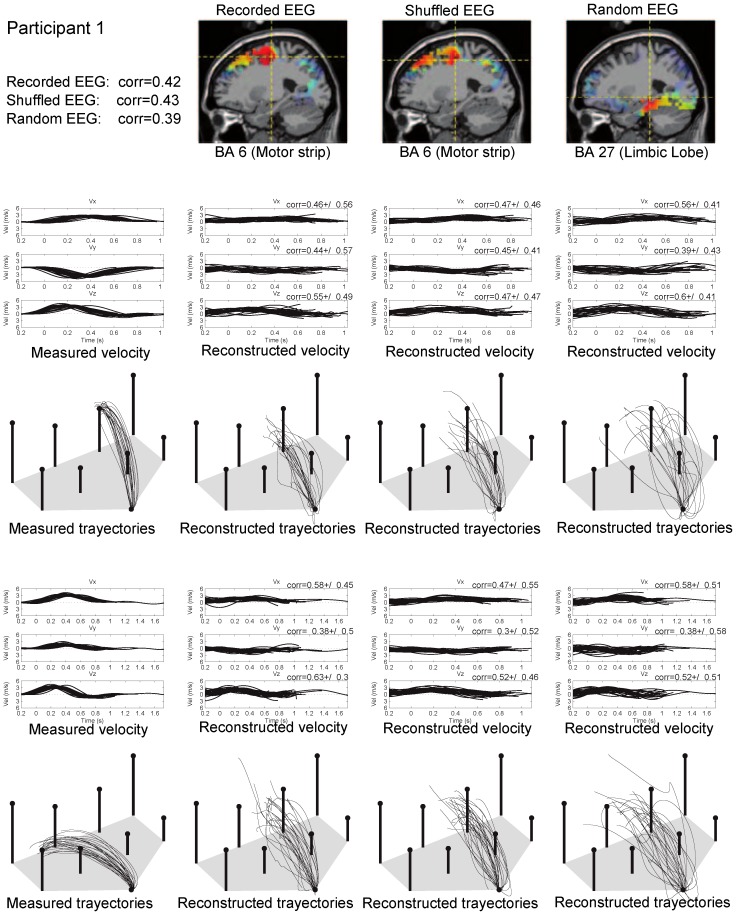
Examples for one of the participants of the source contribution and recorded vs estimated 3D velocity profiles and the corresponding trajectories in two of the targets (obtained with the decoding model that utilizes recorded data, shuffled data and random EEG data). First column displays the measured velocity profiles and trajectories; the second, third and fourth columns display the time course of the reconstructed velocity profiles and the reconstructed trajectories.

Figure 7 shows, for the participant with the best reconstruction results, the source contribution and recorded vs. estimated velocities and trajectories for two of the targets and for decoding models with recorded data, shuffled data and random EEG. In the recorded data and shuffled data decoding models, the precentral gyrus in the frontal lobe (Brodmann Area 6) presented the greatest activation (i.e., direct involvement of the motor cortex), whereas in the random EEG decoding model, the parahippocampal gyrus in the limbic Lobe (Brodmann Area 27) presented the greatest activation (i.e. no direct involvement of the motor cortex). The first column of the figure shows the measured velocity profiles and the corresponding trajectories, while the next three columns show the reconstructed velocity profiles and the corresponding reconstructed trajectories obtained with the recorded data, the shuffled data and the random EEG decoding model, respectively. The reconstructed velocity profiles show that there is little difference between the estimate obtained with the different decoding models and that the mean
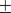
std values of 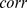 are similar in the three decoding models. In addition, the reconstructed trajectories are similar in the three decoding models. Note that as these trajectories were obtained by integrating the estimated velocity profiles, there is an accumulation of error over time due to the error in the velocity estimation (and then the final target location is never reached). These results show that similar velocity profiles and similar trajectories are reconstructed with the recorded data decoding model and with the chance level decoding models. Analogous results were obtained with other location targets and remaining participants.

#### Progressive Elimination of the Number of Electrodes

**Figure 8:**
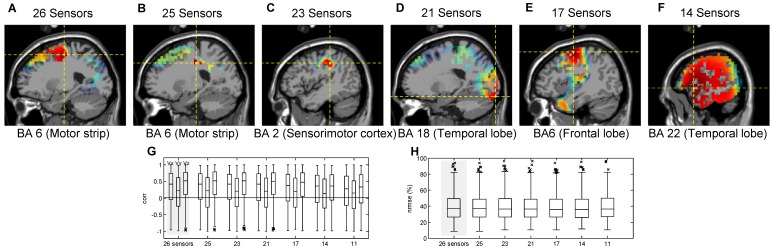
Results of the decoding models for the progressive elimination of the number of electrodes. (A–F) Neural sources involved in encoding hand kinematic projected onto sagittal MRI slices, with dotted lines indicating the source location with the greatest contribution. Contralateral motor regions of the brain provided the greatest contribution in the decoding models with 26, 25 and 23 sensors. No motor related brain region is involved in the other decoding models. (G–H) Distributions of (G) 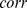 and (H) 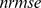 for all participants for decoding models built by progressive elimination of electrodes. These results indicate that significant similar results were obtained in the decoding models that utilize 26, 25, 23, 21 and 17 electrodes, but the results were significantly different and lower when utilizing 14 and 11 electrodes.

For each participant, seven decoding models with 26, 25, 23, 21, 17, 14 and 11 electrodes were built by progressively eliminating the electrodes with major contribution to the regression (see Equation 10). When using the models with higher number of electrodes, the most prominent areas were the motor regions, but the decoding model was forced to use electrodes from other areas as they were progressively discarded. The source contribution analysis showed that in decoding models with 26, 25 and 23 sensors (Figure 8A–C), the precentral gyrus in the frontal lobe (Brodmann Area 6) and the postcentral gyrus in the parietal lobe (Brodmann Area 2) provided the greatest activation, which indicates that the contralateral motor cortex provided the major contribution. In the remaining decoding models, the source contribution analysis showed that no motor region contributes in the decoding (see Figures 8D–F).

Figures 8G and H show the distribution of 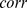 and 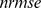 for the seven decoding models. The medians of 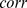 distributions were not significantly different for the first five models (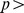
0.05, Kruskal-Wallis test), but were significantly different when utilizing the models with 14 and 11 electrodes. For the latter, the medians of the 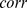 distributions decreased 13, 27 and 17% (in the X, Y, and Z dimensions), and 14, 35 and 20% (in the X, Y, and Z dimensions) in comparison with the model that utilized the entire number of electrodes (26 electrodes). Additionally, the distributions of 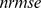 were not significantly different (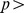
0.05, Kruskal-Wallis test). These results indicated that decoding models with 26, 25, 23, 21 and 17 electrodes obtain significantly similar results using EEG signals from different scalp areas (as the most prominent sensors of each model were progressively discarded), i.e., the same information used in the decoding could be obtained alternatively from different areas of the brain (up to a limit, where if more electrodes are removed the distribution of correlations tends to zero).

**Figure 9:**
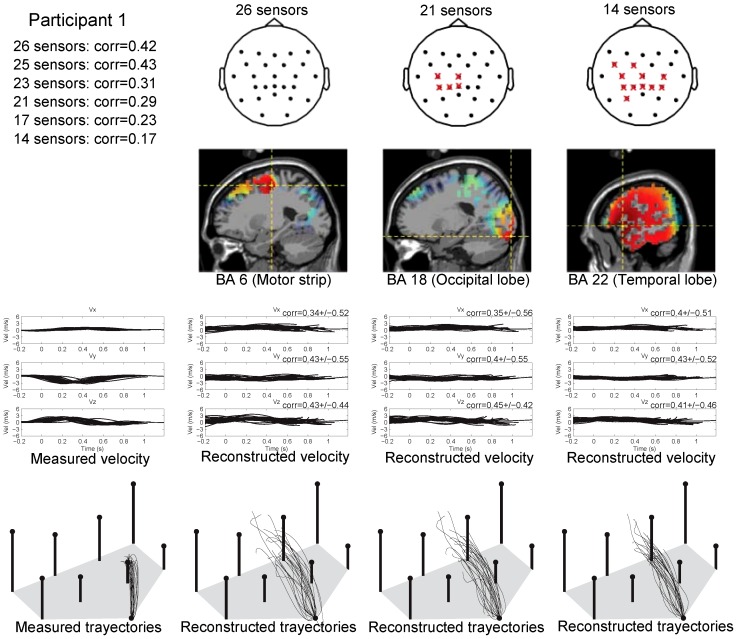
Examples for participant 1 for the decoding model that used recorded data with 26, 21 and 14 sensors. Top: Location of the electrodes (black dots) used to built the decoding model and the removed electrodes (red crosses), and estimated neural sources involved in encoding hand kinematic projected onto sagittal MRI slices. Bottom: Recorded vs estimated 3D velocity profiles and trajectories in one of the targets. The first column displays the measured velocity profiles (upper panel) and trajectories (lower panel); the second, third and fourth columns display the time course of the reconstructed velocity profiles (upper panels) and the reconstructed trajectories (lower panel).

Figure 9 shows examples for participant 1: location of used and removed electrodes and the sources contribution analysis (for the decoding model with 26, 21 and 14 sensors). Note how the electrodes located above the motor strip are removed (red crosses) to obtain the decoding model with 21 and 14 sensors. While in the decoding model with 26 sensors the major cortical contribution was the precentral gyrus of the frontal lobe (Brodmann Area 6) on the left hemisphere (Figure 9 top) involving the motor area, in the decoding models with 21 and 14 electrodes the major cortical contribution was respectively located on the fusiform gyrus in the occipital lobe (Brodmann Area 18) and on the superior temporal gyrus in the temporal lobe (Brodmann Area 22) which are not directly related to the primary motor neural networks. Recorded vs. estimated velocities and trajectories for one of the targets are also displayed in Figure 9. Small differences are observed between the reconstructed velocity profiles obtained with the three decoding models. The mean
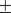
std values of 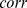 are similar in the three decoding models, and no significant differences were found in the medians of the 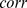 distributions of the three decoding models (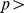
0.01, Wilcoxon rank-sum test).

## Discussion

The interest in reconstructing limb kinematics from EEG is recent, although it is recognized to be a challenge due to inherent difficulties of EEG signals (i.e., low signal-to-noise ratio, limited bandwidth, or poor information content) [10]. However, recent studies have reported achievements using the low frequency activity of the EEG and linear regression models [14]–[19]. This paper analyzed the mathematical properties of the linear regression model and of the correlation metric and how they may affect the interpretation of the decoding results of the analysis. These properties could explain, from a strictly mathematical point of view, the positive correlations when decoding limb kinematics from low frequency temporal signals using linear regression models. However, further investigation was required to verify whether there was a neural correlate behind the trajectory reconstruction using this frequency band or if it was just a misinterpretation of the results of the analysis.

This issue was investigated herein, and this paper reports the result of an experiment where healthy participants performed predefined reaching movements of the hand in 3D space (executed with an average of 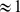
Hz). The first objective was to check whether it was possible to reconstruct the limb velocity profiles from low frequency EEG, with accuracies comparable to the state of the art. The results confirmed that the best reconstruction results were obtained using the (0–1) Hz band, with a positive distribution of the correlations and significantly different from zero for all coordinates. These results were quantitatively of the same order of previous studies [15]–[17], confirming that the best reconstruction of limb velocity profiles occurs when using low frequency activity of the EEG.

The second objective was to understand whether there was a neural correlate behind the reconstruction. This was analyzed by testing the statistical significance of the previous decoding model with chance level decoding models (without physiological relation to the motion process) and with models that progressively eliminated the most prominent sensors (progressive elimination of the physiologic relation to the motion process). On one hand, the correlations and normalized errors of the results of the models were not statistically different (i.e., similar correlations and errors were obtained regardless of whether the decoding model was trained with recorded, shuffled, random EEG or random velocity profiles). Note that shuffled and random data combinations were evaluated to ecover the chance level of the model in the absence of any limb velocity information. On the other hand, when iteratively removing the sensors with major contributions to the decoding (including all those above the motor strip and in the frontal and parietal contra-lateral areas), the results in terms of correlation and normalized errors were not statistically significant. Both results jointly with the source analysis support the fact that, while mathematically there is a solution for each particular dataset, the accuracies of the velocity profiles reconstruction are at chance level (i.e. the model is able to provide the same results irrespectively of the presence or absence of limb velocity information). Also, the same reconstruction accuracy can be obtained by iteratively eliminating the most useful information for the decoding and by using information from other sensors (other brain areas). As a consequence, it is possible to argue that there is no unique information behind the reconstruction in this experiment, and thus it is not possible to claim a decoding with this model.

Note that this result does not mean that low frequency EEG lacks information about movement, rather it only shows that the linear regression model is not able to reconstruct the limb kinematics from low frequency temporal EEG signals (Although not reported in the paper, the same analysis was performed for position profiles leading to the same conclusions). This issue must be highlighted as there are several studies supporting the existence of motor-related potentials (MRP), which are slow shifts in the EEG activity induced by volitional movements [33], [34]. These potentials originate approximately two seconds prior to movement onset developing the Bereitschafts potential (BP) or readiness potential (RP) [35], and rebound during movement execution developing the motor potential (MP). BPs have been used to detect movement intention [36], [37] while MPs have been used to detect movement parameters such as direction [31], [38]. In these approaches the extracted motor information are general movement parameters (i.e., the intention to move, the direction of the movements) usually detected before movement onset or just after movement initiation.

Another possible experiment to understand the decoding model could involve repetitive motions of the limb at different (and higher) frequencies to illustrate how the EEG that better fits the limb kinematics changes with the limb frequency (which will be congruent with the mathematical model). However, any shift in the limb kinematics and the EEG frequencies to evaluate the decoding would not necessarily reveal an absence of neural correlate (i.e., it would be necessary to apply a similar methodology as the one proposed in this paper to evaluate the chance level of the model). This is the reason why this experimental setup was discarded.

The position of this paper with respect to other previous works that describe limb kinematics decoding from low frequency EEG using linear regression models [14]–[19] is that the results must be confirmed to be above chance level. Previous reconstruction claims [17] reported by the authors of the present paper are not valid, as the results of the analysis were misinterpreted. Other studies reported kinematics decoding using linear filtering and EEG activity of higher frequency bands [13], which does not hold the property that both signals must have the same frequency range. However, in this case the noise of the filtering could have played a crucial role, allowing high frequency EEG to fit low frequency velocity profiles based on the ratio of observation and system noise. Moreover, there are several studies that have developed reconstruction of limb movement profiles using invasive recordings of brain activity (ECoG, LFP and SUA). While these studies use linear models, the neural characteristics used in the model are neural firing sequences [8] or non-linear processing of the brain signals [39], [40]. In addition, there are other works where a linear decoding model is used within a biofeedback strategy [1], [3], [27], [41]–[43]. In these cases the subject may learn to self-regulate brain oscillatory activity through internal nonlinear mechanisms that regulate the neural signals utilized by the decoding model (closed loop control). If the decoding model cannot capture all the degrees of freedom of the neural process due to the constraints imposed by the model and the characteristics, then self-regulation would improve the accuracy up to a performance plateau [44]. Finally, the conclusions of this paper cannot be generalized to those studies that address the decoding of individual parameters of motion since these studies use non-linear models, establishing a pattern recognition problem [31], [38], [45] and not a linear regression as studied herein.

There are at least two possible paths to avoid the mathematical constraints of the linear regression in future limb kinematics decodings. One possibility would be to use other characteristics of the EEG signals extracted from the full frequency spectrum or from other frequency bands using nonlinear transformations (e.g. time-resolved power extracted from time-frequency representations [32, 49] or temporal source current estimates [26]). This feature extraction process will, in principle, enable the use of information from different frequency ranges during the reconstruction. Another possibility would be to use a non-linear model to relate the limb kinematics to EEG temporal sequences in other frequency ranges. This would alleviate the constraint that the two sets of temporal signals must present the same frequency ranges.
